# Capsular warning syndrome and its clinical awareness and therapeutic approach: two case reports and a systematic review of the literature

**DOI:** 10.3389/fneur.2023.1177660

**Published:** 2023-05-16

**Authors:** Hector R. Martínez, Jose A. Figueroa-Sanchez, Carlos A. Arreola-Aldape, Jose Alberto Moran Guerrero, Ana Luisa Trujillo-Bracho, Alejandro Cantú López

**Affiliations:** ^1^Tecnologico de Monterrey, Escuela de Medicina y Ciencias de la Salud, Monterrey, Mexico; ^2^Instituto de Neurología y Neurocirugía, Centro Médico Zambrano Hellion, TecSalud, San Pedro Garza García, Mexico

**Keywords:** capsular stroke, capsular warning syndrome, ischemia in internal capsule, burst of focal neurologic events, stroke unit

## Abstract

**Introduction:**

Capsular warning syndrome (CWS) is characterized by recurrent stereotyped episodes of unilateral transient motor and/or sensory symptoms affecting the face and upper and lower limbs, without cortical signs in 24 h and with a high risk of developing stroke. Among the possible underlying mechanisms, small perforating artery disease is the most common. The aim was to assess the most common risk factors, the therapeutic alternatives, and the different outcomes in patients with CWS, along with the presentation of two cases treated in our Emergency Department.

**Methods:**

Stroke Code, launched at our institution in January 2017, was triggered 400 times, and by December 2022, 312 patients were admitted as having an acute ischemic stroke. Among them, two of them fulfilled the criteria of CWS. A systematic search was carried out in PubMed, Scopus, and Web of Science databases to seek demography and therapeutic approaches in CWS.

**Results:**

Of 312 cases, two with acute ischemic stroke exhibited CWS. The first patient had six events of right hemiparesis with recovery in 10–30 min; after MRI and digital subtraction angiography (DSA), he received apixaban and clopidogrel; however, a day after admission, he developed ischemic infarction with partial recovery. The second patient presented five transient events of right hemiparesis. After MRI and DSA with an intra-arterial infusion of nimodipine, oral aspirin, and ticagrelor, he presented another event-developing stroke and was discharged with partial recovery. A systematic review found 190 cases of CWS in 39 articles from 1993 to 2022. Most were male subjects (66.4%), and hypertension (60%), smoking (36%), diabetes (18%), and dyslipidemia (55%) were the most common risk factors. Over 50% of the cases were secondary to small perforating artery disease. The most commonly used treatments were dual antiplatelet therapy (DAT), recombinant tissue plasminogen activator, and anticoagulant therapy (ACT), where the combination of DAT plus ACT was linked to the most positive functional outcomes (82.6%).

**Conclusion:**

Our cases fit with the description of patients with partial recovery and risk factors (hypertension, diabetes, and smoking) in male patients. There is a lack of evidence regarding the best treatment option; dual antiplatelet therapy and anticoagulation therapy are strong contenders for a favorable result.

## Introduction

Capsular warning syndrome (CWS), described by Donnan in 1993, is a pattern of transient ischemic attacks (TIAs) characterized by at least three episodes of unilateral transient motor and/or sensory symptoms affecting two or more regions (face and upper and lower limbs) without cortical signs (aphasia, apraxia, and agnosia) within 24 h (1). CWS has an incidence of 1.5–4.5% of patients with TIA, and its mechanism and treatment remain unclear.

Stroke-related risk factors such as hypertension, diabetes, smoking, and dyslipidemia have been linked to CWS (2). Its exact underlying mechanism remains unknown; nonetheless, small perforating artery disease has been described as the most common cause (3). Atherosclerosis (2), artery-to-artery microemboli (1), and intermittent hemodynamic changes secondary to structural arterial changes or blood pressure fluctuations (4, 5) have also been suggested as potential mechanisms.

Clinical awareness is of utmost importance due to its high risk of developing ischemic strokes with a permanent neurological deficit (6, 7). The 7-day stroke risk following a CWS is as high as 60% (8), with the majority of strokes happening within the first 48 h (7). A variety of treatments have been suggested, including blood pressure control, anticoagulation, antiplatelet therapy, and thrombolytic agents; nonetheless, no definitive approach was described to alter the natural history of this syndrome (1, 2). We report two cases of CWS, a systematic review of the literature was realized to seek evidence regarding risk factors, and the most appropriate therapeutic approach was linked to the best clinical outcomes.

## Methods

In January 2017, the Stroke Code Program was launched at our institution, and until December 2022, this Code was triggered 400 times. In this period, two out of 312 patients admitted with acute ischemic stroke fulfilled the clinical criteria of CWS. These two cases are described along with a systematic review and were performed according to the 2020 Preferred Reporting Items for Systematic Review and Meta-Analysis (PRISMA) guidelines.

The main objective of the systematic review was to identify patients who fulfilled the clinical criteria of CWS in order to assess the most common risk factors, the diverse set of therapeutic approaches [antiplatelet, anticoagulant, and recombinant tissue plasminogen activator (rt-PA)], and the different outcomes described as absent, partial, or complete recovery.

The search was performed on 15 February 2023 in PubMed[Title/Abstract], Scopus [Article title, abstract keywords], and Web of Science [Topic] databases with the following query: capsular warning syndrome. Only articles with case reports and case series published in English and Spanish were considered. The abstracts were manually revised to assess whether they fulfilled the following inclusion criteria: patients who fulfilled the clinical criteria of CWS (≥three TIAs in <72 h), without age, gender, or comorbidities restrictions, who received pharmacological treatment with either antiplatelet, anticoagulant, rt-PA, or a combination of the drugs, and whose treatment's efficacy and clinical outcome were assessed.

**Figure 1:**
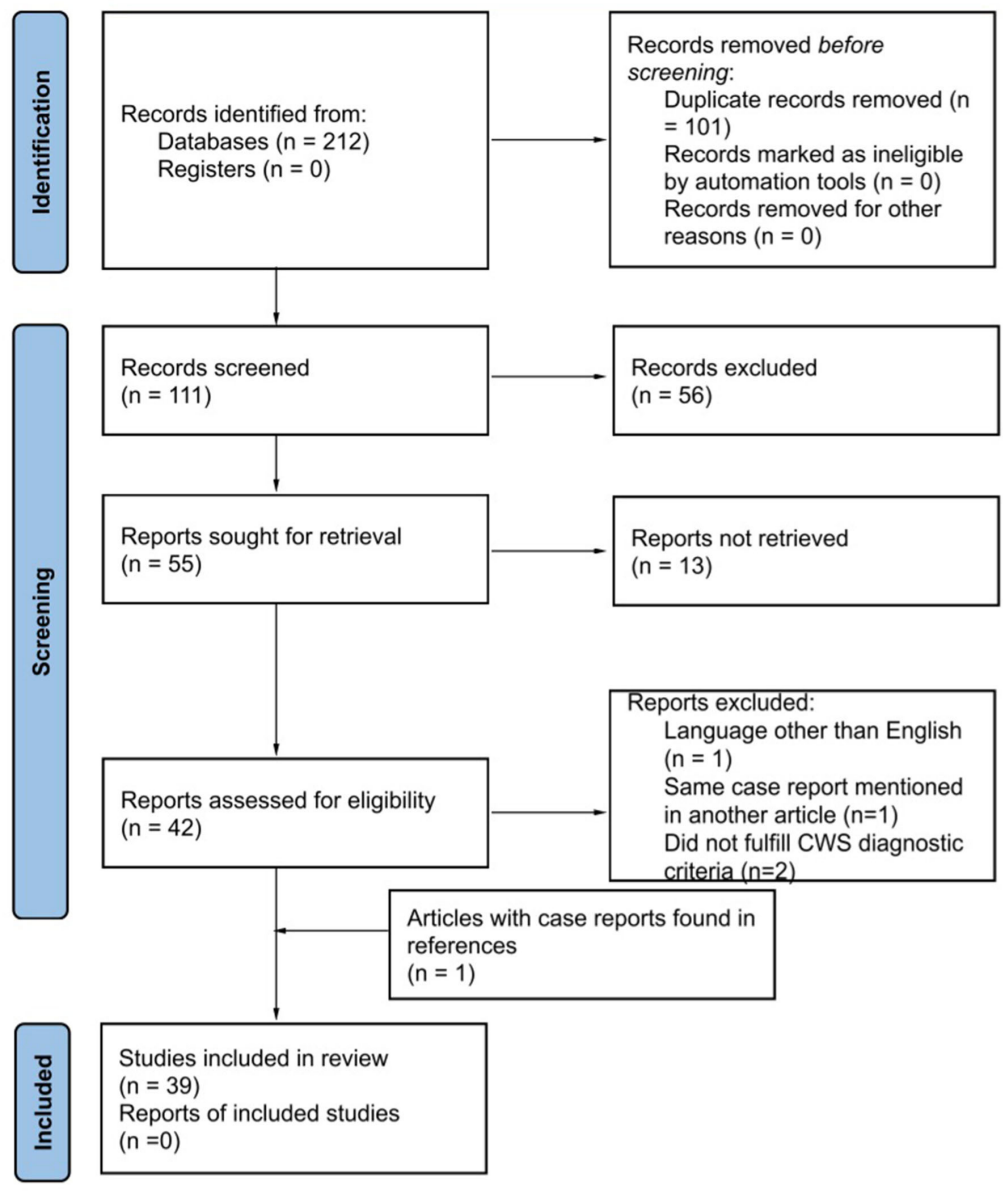
Identification of studies via databases and registers. Preferred Reporting Items for Systematic Review and Meta-Analysis (PRISMA) 2020 flow diagram for systematic reviews.

Among these databases, 111 articles were screened, and 56 were excluded as they were unrelated, described pontine warning syndrome and not CWS, or did not include a case report. Finally, 42 articles were assessed for eligibility, and 38 fulfilled the inclusion criteria. Within the citations of our retrieved articles an additional case report that met the inclusion criteria was found, adding up to 39 studies included in the analysis (Figure 1).

The present study adds a compilation of 190 cases of CWS dating from 1993 to 2022. Two authors realized the data extraction individually and compared it to reduce extraction errors. All discrepancies were reviewed by a vascular neurologist and a vascular surgeon (HRM/JAFS). The following data were included: patients' characteristics, including demographic information (age, sex, and risk factors such as smoking, drinking, current alcohol use, and drug use), CWS criteria according to the authors, episodes of TIAs, treatment, and outcomes. Outcome was defined as “recovery” (resolution of symptoms and regain of function), “partial recovery” (partial resolution of symptoms and partial regain of function described as a clinical amelioration during the neurological examination or NIHSS score improvement at discharge), and “no recovery” (no symptom resolution or amelioration at discharge). The demographic and clinical characteristics were analyzed using Microsoft Excel (Microsoft Corp., Redmond, WA, USA).

## Results

### Representative cases

The following cases were evaluated in our stroke unit. CWS diagnosis was made through a thorough neurological evaluation in the emergency room after imaging studies were performed. All other causes of focal neurological deficit, such as epilepsy, brain tumors, cerebral hemorrhages, concussions, migraine, psychogenic paralysis, and transient global amnesia, were excluded from the registry as well as those patients with the diagnosis of one isolated event of TIA.

**Figure 2:**
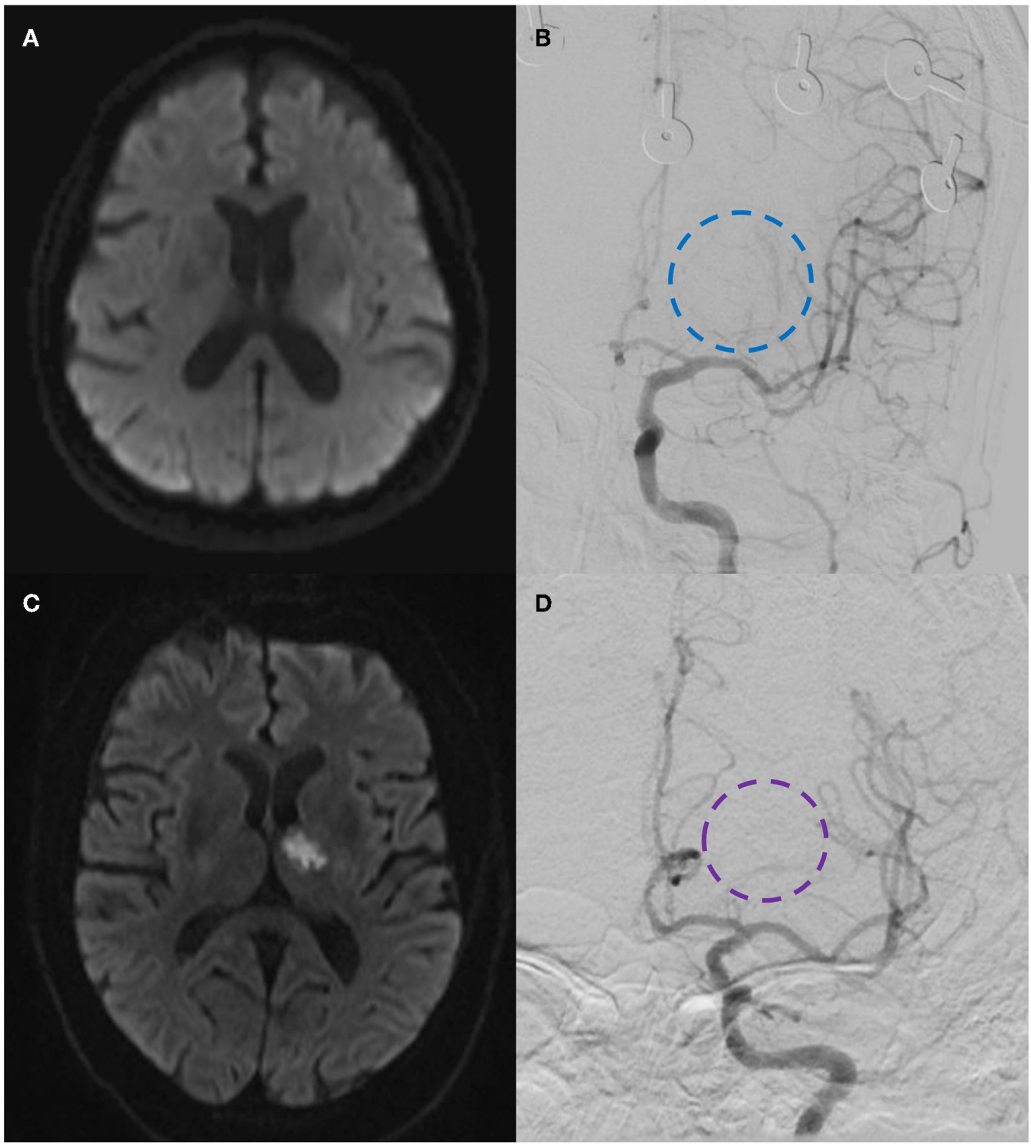
Patient 1: **(A)** Diffusion-weighted magnetic resonance imaging (DW-MRI) showing a subtle hyperintense signal in the left internal capsule and thalamus. **(B)** DSA showing hypoperfusion of the medial and lateral lenticulostriate penetrating arteries (dashed-blue circle). Patient 2: **(C)** DW-MRI showing a hyperintense signal in the left thalamus and internal capsule. **(D)** DSA showing a decreased vascular perfusion in the lenticulostriate penetrating arteries of the left middle cerebral artery (dashed-purple circle).

*Patient 1:* A 59-year-old man with a history of diabetes, hypertension, and smoking arrived at our emergency room after six events throughout 48 h of right hemiparesis lasting 10–30 min each. Four events happened a day before in Spain and during his flight back to Mexico, and two additional transient episodes of right hemiparesis and numbness happened before his arrival at the Emergency Department. The patient was alert in the emergency room with normal speech and neurological examination. The MRI showed a subtle hyperintense signal in a diffusion-weighted image with normal T2 and FLAIR-weighted images. As CWS was among the differential diagnosis, a digital subtraction angiography (DSA) was performed. The DSA showed hypoperfusion of the medial and lateral lenticulostriate penetrating arteries. Embolic and thrombotic etiologies were ruled out with a transesophageal echocardiogram, carotid ultrasound, and Holter monitor; hypercoagulable states and underlying rheumatologic diseases were ruled out through lab testing. He was admitted to the intensive care unit and received apixaban, clopidogrel, and antihypertensive and antidiabetic drugs. A day after, he presented another event of right hemiplegia with normal speech. A follow-up MRI at 24 h was performed and revealed an ischemic infarction in the left internal capsule and left thalamus (Figures 2A, B). No additional events were witnessed. He was discharged 2 weeks after admission with a partial recovery described as persistent right hemiparesis without any other neurological deficit (CARE Checklist and Flow Diagram as Supplementary material 1A, B).

*Patient 2:* A 69-year-old man with a history of smoking and hypertension presented a right hemiparesis after having sex early in the morning with complete recovery in 15 min; he had two similar events on the same day with a full recovery. After arrival at the emergency room, he had another right-sided weakness episode with a normal speech of 5 min duration. The neurologist found an alert patient with normal speech without motor or sensory abnormalities. The diagnosis of transient ischemic attack was established. During his transfer to MRI, he presented another episode of right hemiplegia; at this time, the neurological examination revealed right hemiparesis, normal speech, and lower facial paralysis. Hoffman and Tromner reflexes were present, and the NIHSS score was 7 points. The MRI revealed a hyperintense signal in the left thalamus and internal capsule in a diffusion-weighted image with normal T2 and FLAIR-weighted images (Figures 2C, D). The patient was transferred to DSA, which showed decreased vascular perfusion in the lenticulostriate penetrating arteries of the left middle cerebral artery. The diagnostic workup was the same as for patient 1 to rule out other etiologies and differential diagnoses. After a super selective intra-arterial infusion of 10 mg nimodipine, the parenchymal perfusion improved, and he showed complete recovery of right hemiparesis. He received aspirin and ticagrelor and antihypertensive drugs. However, the patient presented another episode of right hemiparesis a day after without clinical recovery. He was discharged 10 days after hospitalization with right upper and lower limb paresis (2/5), alert with normal speech (CARE Checklist and Flow Diagram as Supplementary material 2A, B).

### Systematic review

**Figure 3:**
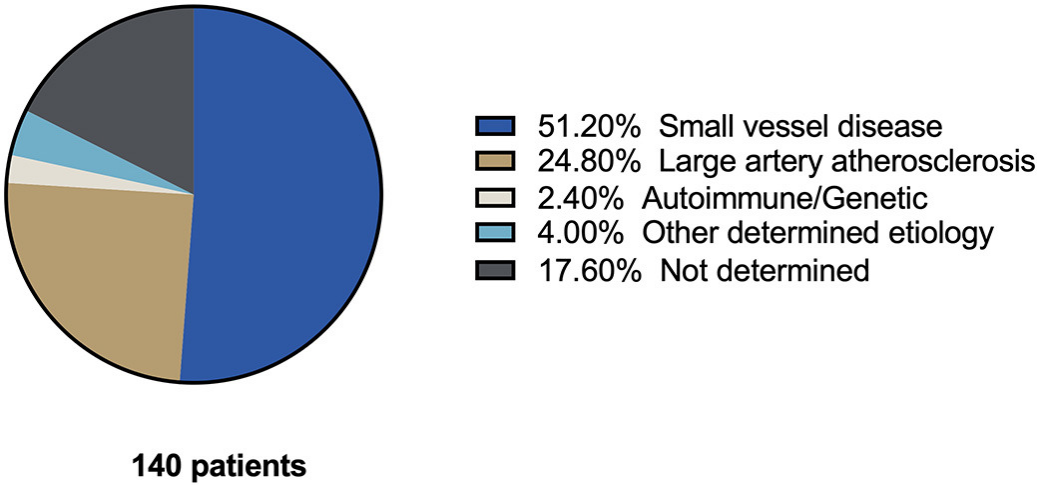
Etiology of capsular warning syndrome cases found in the literature. Pie chart of etiologies reported in 140 CWS cases found in our systematic review of the literature.

From the 39 included articles found in the database search (Figure 1), 190 CWS cases described in the literature from 1993 to 2022 were submitted to this systematic review, and 140 were used in the analysis (Table 1). Most patients were male subjects (66.4%) and above 50 years old. Arterial hypertension was the most common risk factor (60%), followed by dyslipidemia (55%), smoking (36%), and diabetes (18%). A total of 20 patients did not have any risk factors. Considering the available data, over 50% of CWS cases were attributed to small vessel disease (Figure 3), and 55% of the cases presented in the review progressed into an ischemic stroke (Table 1).

**Table 1:** Studies found describing CWS cases and their characteristics.

										**Treatment**				
**Reference**	**Total cases**	**Sex (male, female)/ age (years)**	**Stroke risk factors** ^*^	**Mean events/ CWS hours**	**CWS NIHSS**	**Stroke**	**Stroke NIHSS**	**CT**	**MRI**	**DAT/SAT/ ACT** ^**^	**rt-PA**	**Outcome** ^***^	**Follow-up**	**Suspected etiology** ^†^
Payus et al. (6)	1	M/62 y	SM	8/12 h	-	No	-	1	0	DAT	0	R	R	SVD
Baharnoori et al. (9)	1	M/22 y	None	4/1.5 h	-	Yes	-	1	1	ACT	0	PR	Deficit (2 m)	Cannabis-related stroke
Shen and Heshmati (10)	1	M/3 y	None	4/5 h	-	Yes	8	1	1	SAT	1	PR	R (3 m)	Post-VZV artheriopathy
Nadarajan and Adesina (11)	1	M/72 y	HTN, SM, DM	7/36 h	-	Yes	-	1	1	DAT	0	PR	Deficit (6 m)	SVD
Cohen et al. (12)	1	M/58 y	HTN, SM, DL	2/7 h	10	Yes	-	1	0	DAT	0	PR	Mild paresis (4 m), Stable (14 m)	Atherosclerosis of RAH
Bonardo et al. (13)	1	M/39 y	None	11/72 h	-	Yes	-	1	1	DAT	0	R	-	M2 dissection
Romero and Ortiz Salas (14)	1	M/72 y	None	6–11/72 h	-	Yes	-	1	1	SAT, ACT	0	PR	-	Antiphospholipid syndrome
Jiao et al. (15)	1	F/63 y	None	4/24 h	4	No	-	0	0	DAT, ACT	0	R	R (3 m)	AchA stenosis
Chen et al. (16)	1	M/47 y	SM	5/24 h	-	Yes	-	0	1	DAT	0	PR	mRS 1 (6 m)	MCA dissection
Caporale et al. (17)	1	M/57 y	None	6/128 h	-	Yes	-	0	0	SAT	0	PR	R (12 m)	Thin AchA
Farrar and Donnan (18)	1	M/72 y	HTN, DL	16/24 h	-	Yes	-	0	0	ACT	0	PR	Improvement (3 w)	SVD
Colla Machado et al. (19)	1	M/57 y	SM	5/24 h	6	No	-	1	0	SAT	1	R	mRS 0 (1 m)	SVD
Barral et al. (20)	1	F/83 y	HTN	“Several”/ 48 h	-	No	-	1	1	DAT	0	R	R	M1 stenosis
Teng and Hong (21)	1	M/66 y	None	6/48 h	11	No	-	0	1	SAT, ACT	0	R	R	Atherosclerosis
Benito-Leon et al. (22)	1	M/59 y	None	9/48 h	-	Yes	-	0	1	ACT	0	R	R (3 m)	SVD
Ferro et al. (23)	1	M/44 y	SM	3 or more/48 h	-	No	-	0	1	SAT, ACT	0	R	-	Thrombophilia
Kawano et al. (24)	1	M/66 y	HTN, DL	13/48 h	-	Yes	-	0	1	DAT, ACT	0	R	-	SVD
Tang et al. (25)	1	M/55 y	SM	4/24 h	-	Yes	-	0	1	DAT	0	R	R	Microscopic Polyangiitis
Xu et al. (26)	1	M/47 y	HTN, SM	6/24 h	6	Yes	12	1	1	DAT	1	R	mRS 0 (3 m)	M1 stenosis
Fuseya et al. (27)	1	F/70 y	HTN, DL, DM	5/24 h	3	Yes	9	0	1	DAT, ACT	1	PR	mRS 2 (1 m)	SVD
Springer and Labovitz (28)	1	F/79 y	None	“Several”/ 24 h	-	Yes	-	0	1	None	1	PR	Subtle paresis (3 m)	SVD
Bain et al. (29)	1	M/63 y	HTN, SM	4/24 h	6	Yes	8	1	0	SAT	1	PR	mRS 0 (3 m)	SVD
González Hernández et al. (30)	1	M/75 y	HTN, SM	3/24 h	5	Yes	9	1	0	None	1	PR	NIHSS 1 (Discharge)	SVD
Gutiérrez Ruano et al. (31)	1	M/52 y	1 SM	4/24 h	4	Yes	11	0	2	None	1	PR	mRS 3 (Discharge)	SVD
Asil et al. (32)	2	1M, 1F/64 y	1 HTN	7 or more/24 h	-	2	-	2	2	2 DAT	0	2 R	-	2 SVD
Kamo et al. (33)	2	2 M/46.5 y	1 HTN, 1 DL, 2 DM	>10/24 h	4	1	-	0	2	2 DAT, 1 ACT	0	1 R, 1 PR	-	IC and MCA stenosis
Oliveira-Filho et al. (34)	2	2F/67.5 y	None	5.5 or more/24 h	-	2	-	2	2	1 SAT, 2 ACT	0	1 R/1 PR	-	2 SVD
Fahey et al. (35)	2	1M, 1F /63.5 y	2 HTN	13 or more/48 h	-	1	-	0	2	2 DAT, 1 ACT	0	2R	mRS 0 (3 m)	1 SVD, 1 Basilar Atherosclerosis
Xue et al. (3)	2	2M/47 y	2 HTN, 2 SM, 2 DL, 2 DM	8/24 h	7.5	2	9	2	2	2 DAT	2	2 R	mRS 0-1 (Discharge)	2 SVD
Zhou et al. (36)	2	1M, 1F/58.5 y	1 HTN	7.5/24 h	11	2	3	2	2	2 DAT	0	2 PR	NIHSS 1 (Discharge)	2 MCA Atherosclerosis
Vivanco-Hidalgo et al. (37)	4	3M, 1F/67.5 y	3 HTN, 1 SM, 3 DL, 1 DM	4/6.7 h	-	4	10.2	4	4	None	4	3 R/1 PR	3 mRS 0 (discharge)	4 SVD
Marsh and Llinas (38)	7	4M, 3F/59.5 y	5 HTN, 5 SM, 2 DL, 3 DM	Not provided	3.7	4	-	0	7	7 DAT	0	4 R/3 PR	-	5 SVD
Staaf et al. (39)	8	3M, 5F/73 y	3 HTN, 1DM	5.3/24 h	-	7	-	0	8	4 SAT, 6 ACT	0	4 R/4 PR	-	7 SVD
Sundar et al. (40)	9	6M, 3F/31 y	1 HTN	5.3/25.2 h	5.7	6	-	9	7	8 DAT, 1 SAT + ACT	5	6 R/1 PR/2 NR	-	6 SVD, 3 MVO
Liu et al. (41)	20	15M, 5F/61.6 y	17 HTN, 13 SM, 17 DL, 7 DM	4 or more/24 h	-	20	8.9	20	20	20 DAT	20	6 R/13 PR/1 NR	19 mRS ≤ 2 (3 m)	13 SVD
Li et al. (42)	23	17M, 6F/58 y	14 HTN, 10 SM, 17 DL, 2 DM	4.7/24 h	7.7	10	8.8	23	23	14 DAT, 3 SAT, 6 ACT	5	16 R/7 PR	22 mRS ≤ 2 (3 m)	19 Cerebral Atherosclerosis
Foschi et al. (7, 43)	33	18M, 15F/64.8 y	25 HTN, 9 SM, 32 DL, 6 DM	3/72 h	-	10	4	33	11	16 DAPT, 14 SAPT, 3 ACT	6	23 R/9 PR/1 NR	32 mRS ≤ 2 (3 m)	10 SVD
Donnan et al. (1)	50	31M, 19F/63 y	42 HTN, 19 SM, 9 DL, 5 DM	6.1/3 h to 4 days	-	21	-	25	0	36 SAT, 7 ACT	0	21 PR/3 NR	-	15 SVD

For studies presenting 2 or more cases, age, events, NIHSS, and CWS hours are expressed as the mean of overall cases. ^*^HTN, hypertension; SM, smoking; DL, dyslipidemia; DM, diabetes. ^**^DAT, dual antiplatelet therapy; SAT, single antiplatelet therapy; ACT, anticoagulant. ^***^R, recovery; PR, partial recovery; NR, no recovery. ^†^RAH, recurrent artery of Heubner; AchA, anterior choroidal artery; IC, internal carotid; MCA, middle cerebral artery; M1, M2, M1 & M2 segments of middle cerebral artery; mRS, modified Rankin scale; NIHSS, National Institutes of Health Stroke Scale; SVD, small vessel disease; MVO, mayor cerebral vessel occlusion.

Initial treatment (Table 1), given at the first point of care by a medical practitioner upon arrival to the Emergency Department, varied among the reports found in the literature. The different approaches found were heterogenous as antiplatelet drugs, anticoagulant therapy, and thrombolysis were used alone or in combination and, in some cases, were followed by secondary stroke prevention therapy.

**Figure 4:**
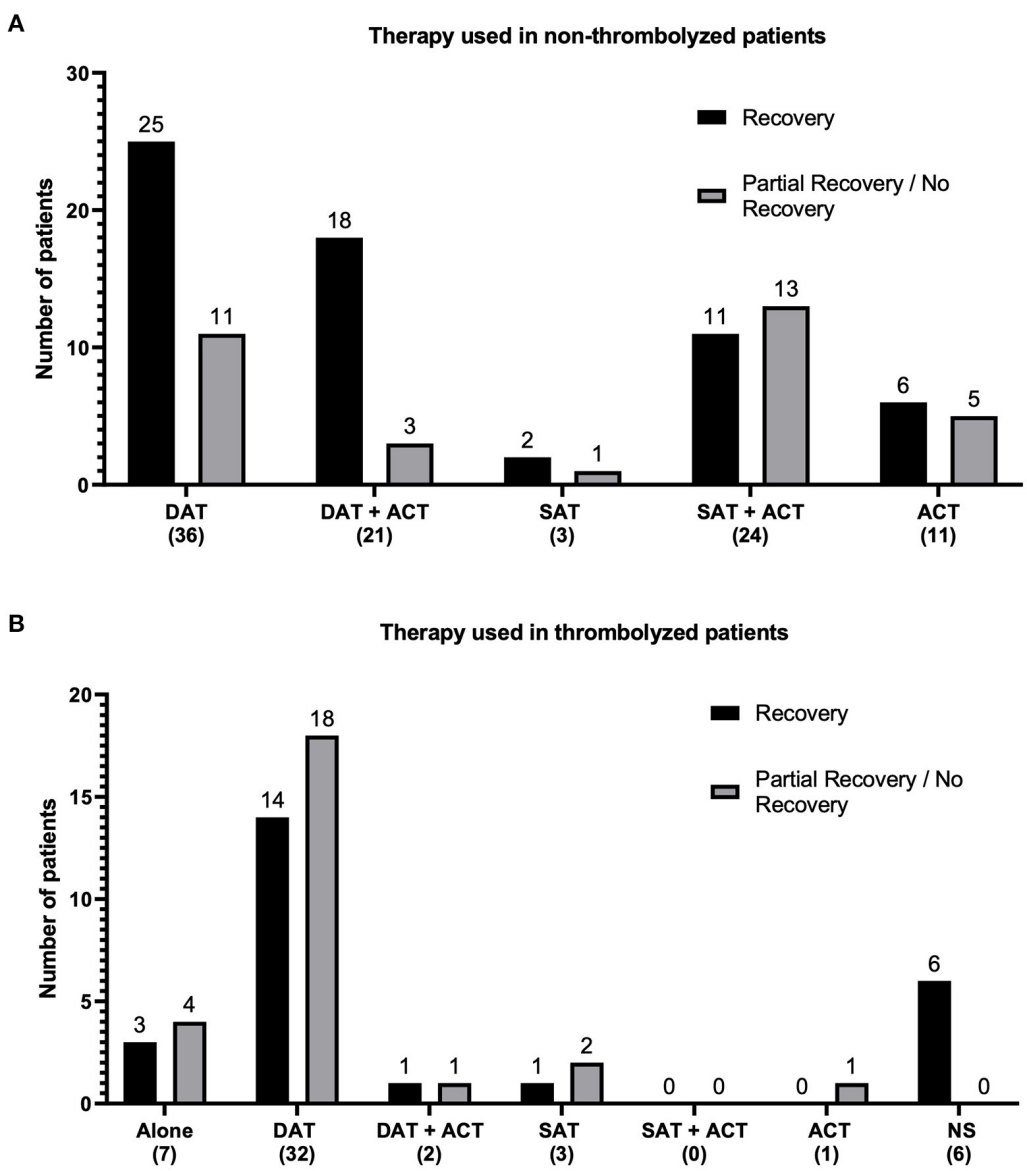
Summary of antithrombotic therapies reported in the literature. Bar graphs **(A)** of therapies used in patients who did not receive thrombolysis with rt-PA and **(B)** therapies used in patients who arrived within the thrombolysis therapeutic window and received rt-PA. Frequencies of the different combinations of antiplatelet and anticoagulant drugs reported in the literature search are plotted in the vertical axis. The therapy combination and total number of patients receiving such combination are plotted on the horizontal axis. Fully recovered (black columns) and partially recovered/unrecovered patients (gray columns) are denoted. DAT, dual antiplatelet therapy; SAT, single antiplatelet therapy; AC, anticoagulant; NS, not specified antiplatelet therapy.

Antiplatelet drugs—single (SAT) or dual (DAT)—were given to 121 patients (SAT: 30, DAT: 91), and 59 cases received anticoagulant therapy (ACT). In 63.7% of patients treated with DAT and 61% with ACT, recovery was described. A total of 23 patients treated with DAT plus ACT had recovery in 82.6% of the cases. There were more patients treated with dual antiplatelet therapy (91) than single antiplatelet therapy (16); however, partial recovery was reported in 36.2 and 53.3% of cases, respectively. The previous results are summarized in Figure 4A.

A total of 51 patients received rt-PA treatment; thrombolysis alone was applied in seven patients, and 44 patients received rt-PA associated with other drugs, such as DAT (18), DAT plus ACT (2), and SAT (3) without specified antiplatelets (6) and with ACT (1). From the 51 patients treated with rt-PA alone or combined, 3 and 22 showed recovery, respectively (Figure 4B). Most patients who received thrombolysis initiated antiplatelet drugs as secondary prevention 24 h after rt-PA (3, 10, 19, 29, 40). Two patients received antiplatelets before thrombolysis (26, 27). One study used a tirofiban infusion within 24 h of thrombolysis (41), while another study used an initial tirofiban infusion followed by thrombolysis for cases showing poor response to tirofiban treatment (42).

## Discussion

CWS consists of identical recurrent TIAs within 24 h with face and arm and leg paresis without cortical symptoms (1); nonetheless, a heterogeneity of diagnostic criteria exists as several authors consider the diagnostic time frame as less than 72 h (7, 44). The absence of cortical signs is due to the confined injury of the internal capsule, or in some cases, vascular damage in the pons, midbrain, or thalamus (1, 44). Pure motor hemiparesis affecting the face, arm, and leg with normal speech is a characteristic of CWS (4). Our two cases showed this clinical presentation that evolved into hemiplegia, even after treatment with DAT, and one of them after super selective intra-arterial nimodipine infusion into the left lenticulostriate arteries.

There are various underlying mechanisms that are suggested to elucidate the pathogenesis of CWS, and the most common cause of this syndrome is small perforating artery disease (3), which was the attributed cause of CWS in our patients. Arterial hypertension that generates lipohyalinosis and endothelial dysfunction that occurs in diabetic patients were among the most common risk factors and probably associated with vasculopathy in lenticulostriate arteries producing the CWS. Previous TIAs have been associated with a positive early outcome in non-lacunar ischemic stroke; nonetheless, this possible phenomenon of ischemic tolerance has not been seen in small vessel ischemic disease (45). Our patients with CWS fit the description of cases that showed partial recovery and the incidence of hypertension and smoking as risk factors, predominantly in male subjects (1, 46–48). One of our patients also presented with type 2 diabetes.

Intravenous thrombolysis, oral anticoagulants, and vasopressors have been used to treat patients with CWS. There has not been an agreement on which treatment is the best and regardless of the various available ones being used, we still do not have enough evidence to see if these affect the natural history of CWS (1, 2, 6). In the present series, the first patient presented six recurrent events of right-sided hemiparesis before developing an ischemic stroke despite receiving antiplatelet drugs and oral anticoagulants. The second patient presented five events of right hemiparesis. In the fifth ischemic event of longer duration, he returned to normal baseline after super selective cerebral intra-arterial nimodipine during the DSA. However, a day after, he developed an ischemic stroke while being under treatment with dual antiplatelet drugs. Our cases showed a partial recovery.

A retrospective study reported that using dual antiplatelet therapy associated with anticoagulant therapy decreased clinical fluctuations and improved functional outcomes showing complete recovery in 85% of cases. A different study highlights intravenous tirofiban infusion, where all 15 patients reached a favorable outcome (42). These treatments are potential options since it has been reported that elevating the blood pressure will reduce distal vessel hypoperfusion, thereby improving perfusion to the affected areas and reducing the risk of adverse outcomes (11). The rt-PA has been associated with a favorable outcome in 26 out of 43 patients where rt-PA was used alone or combined with anticoagulant and dual antiplatelet drugs.

Overall, CWS seems to be related to small vessel occlusion (SVO) due to endothelial dysfunction secondary to diverse mechanisms. According to the results, whenever in the thrombolysis therapeutic window, thrombolysis alone or in combination with DAT might be used. Evidence suggests that even patients receiving direct oral anticoagulant therapy can safely receive intravenous thrombolysis (49). However, DAT and DAT plus anticoagulant therapy could possibly be effective therapeutic options. Patients who received prompt treatment, either with rt-PA, DAT, or DAT plus ACT, had a tendency toward a functional recovery at their follow-up visits (Table 1).

Drugs that improve endothelial function (50), the effects of using rt-PA, antiplatelets, and anticoagulants in CWS, and the therapeutic administration routes should be studied in a multicenter clinical randomized trial to assess its benefits in the CWS.

### Limitations

We found in this systematic review that gathering certain variables was a difficult task since they were not mentioned, or translation was unavailable. The diagnostic criteria, especially regarding the time cohort, vary among the authors; thus, a time frame of <72 h was considered for the analysis. As there is limited information regarding CWS, case reports were included in the analysis and were filtered to fit the criteria for our study; therefore, it turned into a smaller sample size, which might have affected the results.

## Conclusion

CWS is a rare clinical syndrome with a high risk of developing ischemic stroke. The pathophysiology and effective treatment of CWS remain unclear; dual antiplatelet therapy and anticoagulation therapy are strong contenders for a favorable result.

## Data availability statement

The raw data supporting the conclusions of this article will be made available by the authors, without undue reservation.

## Author contributions

HM: supervision, project administration, and writing—reviewing and editing. JF-S: methodology and writing—reviewing and editing. AT-B: writing—original draft and investigation. CA-A: writing—reviewing and editing and investigation. JM: formal analysis. AC: writing—original draft. All authors contributed to the article and approved the submitted version.
