# Supplementary figures and images for: Capsular warning syndrome and its clinical awareness and therapeutic approach: two case reports and a systematic review of the literature
Source: Front Neurol. 2023 May 16;14:1177660. doi: 10.3389/fneur.2023.1177660 (PMC10227623; doi:10.3389/fneur.2023.1177660)

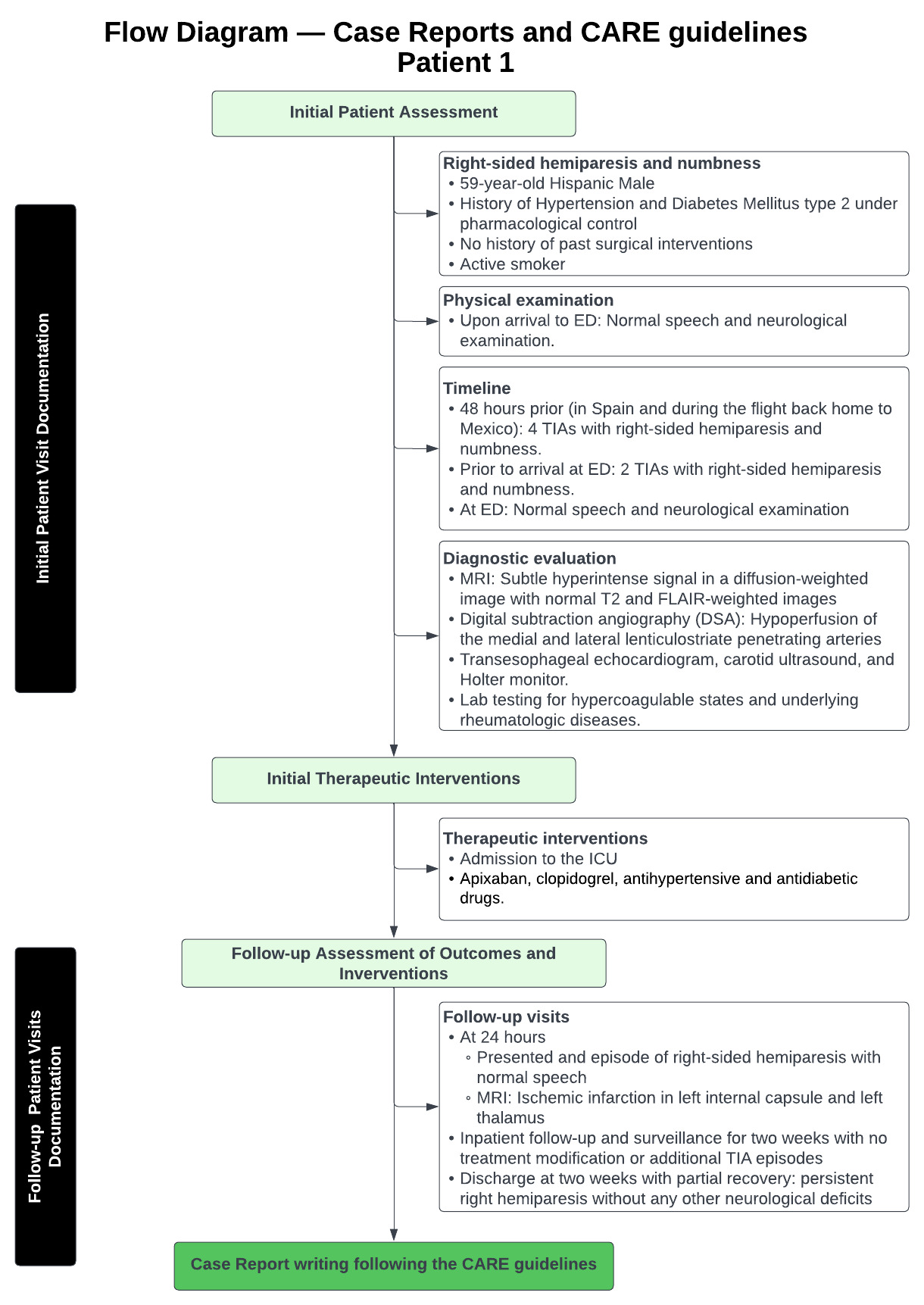

Supplement: Supplementary file 1 [file Image_1.JPEG]

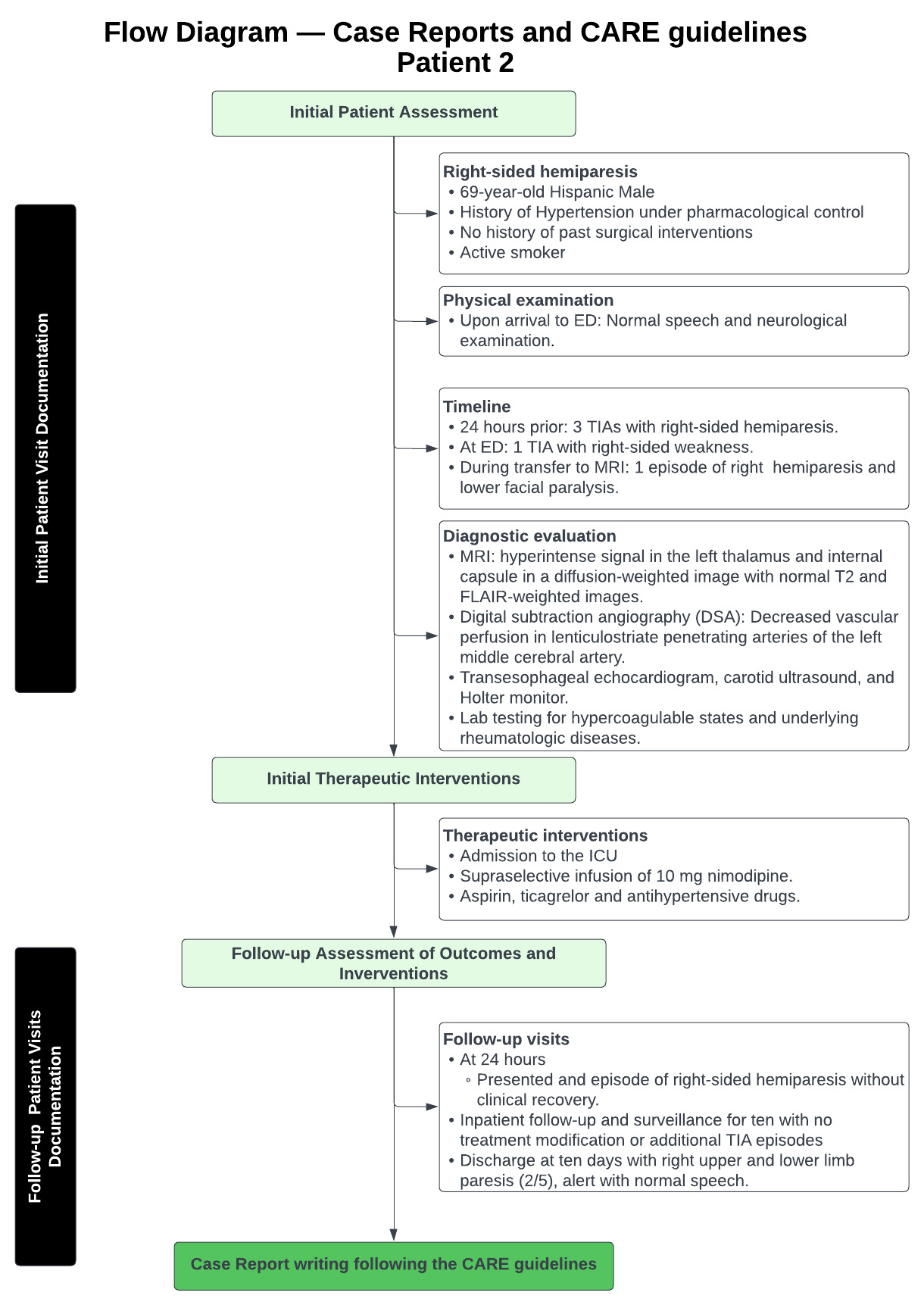

Supplement: Supplementary file 2 [file Image_2.JPEG]
